# Doctors-in-training support strategy from incident report point of view

**DOI:** 10.1016/j.amsu.2020.06.032

**Published:** 2020-06-26

**Authors:** Tatsuya Fukami, Masakazu Uemura, Yoshimasa Nagao

**Affiliations:** Department of Patient Safety, Nagoya University Hospital, 65 Tsurumai-cho, Showa-ku, Nagoya, 466-8560, Aichi, Japan

## 1 Introduction

Medical doctors need continuous patient safety education from medical student. With respect to patient safety, reduced the adverse event for the patient [1]. However, doctors-in-training and other doctors need different approaches. “To Err Is Human,” published by the Institute of Medicine in 1999 [2], is a landmark study in global patient safety as well as the United States healthcare system. The report encouraged highly effective interventions that have since been developed and adopted for hospital-wide incidents including near-miss and adverse events from incident reports. James Reason has suggested that safety culture consists of four elements [3], namely reporting culture, learning culture, just culture, and flexible culture, among which reporting culture is the essential component. An incident-reporting system is a voluntary, anonymous, confidential electronic system that allows the reporting of incidents and adverse events for analysis by experts in quality improvement and patient safety [4,5]. The purpose of this study is to identify the higher-risk aspects of incident data obtained from the hospital-wide reporting system and to establish which factors are related to improvement measures and recommendations that significantly reduce near-miss or adverse events in doctors.

## 2 Material & method

Patient safety incident reporting is mandatory for all staff in our hospital, including contracted workers, when they confront an incident. We surveyed the incident reports submitted from fiscal years 2015–2018, selected from all incidents reported by all hospital workers, and collected information from the corresponding original electronic incident reports. We compared the reports by medical doctors and other workers in the hospital. The collected data included: the date of the incident; ward/department where the incident occurred; healthcare profession, years of experience, and affiliated department of the reporter and person involved in the incident; information regarding the patient; incident details; incident classification; and incident severity classification. Incident severity classification is widely used to evaluate the impact on the patient conveyed by the incident. Near-miss, whereby an unexpected event has the potential to cause but does not actually harm the patient or interrupt the normal situation. A near-miss is often an error prevented by other circumstances. Adverse event, which represents any unintentional or unfavorable clinical sign or symptom, including complications, any new illness or disease or the deterioration of existing disease or illness, and any clinically significant deterioration in any laboratory assessments or clinical tests [6]. The electronic incident reporting system used by our hospital is the Incident Report System version 1.0 (Safe Master, Fukuoka, Japan). We extracted only necessary incident information items for this study, and processed information concerning individuals (e.g., the reporter and target patient) anonymously. About patient and public involvement statement, this study was approved by the Institutional Review Board of the study hospital. Registered to Research Registry Unique Identifying Number is researchregistry5734 [7]. When patient safety incidents and accidents occur in the hospital, they are submitted to the general risk and safety managers from various occupations via an electronic reporting system.

## 3 Result

**Fig. 1:**
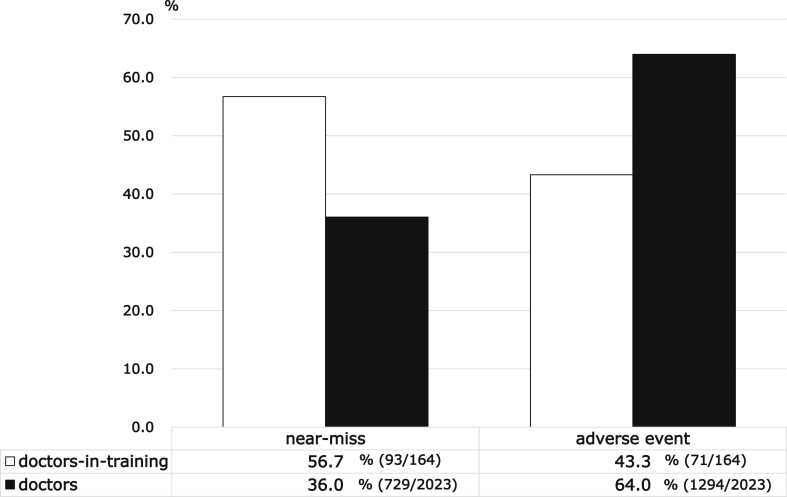
Distribution of incident severity reported by medical doctors and doctors-in-training.

We describe the previous report as the incidents reported by medical doctors are important and represent the organization's transparency [8]. The incidents with higher impact on patients significantly increased the number of reports by medical doctors [8]. Doctors-in-training (postgraduate year 1 or 2) tend to more frequently report near-miss events in comparison with other doctors (Fig. 1). There is a significant difference between the groups. This study shows the fundamental support needs for doctor-in-training.

## 4 Discussion

Increased direct attending physician supervision did not significantly reduce the medical error rate [9] and over supervision and its effect on hindering trainee competence and longer-term patient safety. Organization instructors should educate for the doctors-in-training, what they need to know the safety culture and patient safety is first, how to read the instruction of medical electronic devices and the attached document of drugs, how to prevent and recover from adverse events and know their limitations on what they can or not and the medical team competencies with team training [10]. And organization constructs how to educate and evaluate the basic medical knowledge and procedures for the clinical privilege. Practice the incident reporting to improve the quality of healthcare [11]. Adjustment needs between supervision and autonomy. The incident reporting system is a worthwhile source of information from which to discover potential risks and attributable factors of a representative patient safety issue [4,12]. Accumulation of near-miss incidents of the same type and with a small impact as a one-off event also carries the risk of potential adverse events. In the present study, doctors-in-training reported a higher percentage of near-misses than adverse events, because they are not wholly familiar with procedures and systems. Doctors in-training should thus be offered support to allow them to feel comfortable in their working environment.

## 5 Conclusion

In conclusion, reporting of incidents by medical doctors reflects the patient safety and quality improvement in healthcare. Appropriate intervention for patient safety can be performed depending on the target.

## Ethical approval statement

In order to keep the ethical soundness of the research, an ethical approval letter was obtained from the Institutional Review Board (IRB) of Nagoya University Hospital. Comprehensive consent was also secured before data collection. (No. 2018-0283).

## Funding

The author disclosed receipt of the following financial support for the research, authorship, and/or publication of this article: This work was supported by Terumo Life Science Foundation (Kanagawa, Japan).

## Author contribution

Participated in the conception and design of the study: TF. Analyzed the data: TF, MU. Collected data: TF, MU, YN. Interpreted the data: TF. Drafted the initial manuscript: TF, YN. All the authors have read, contributed to and approved the final manuscript.

## Registration of research studies

Researchregistry5734. https://www.researchregistry.com/browse-the-registry#home/?view_2_search=5734&view_2_page=1

## Guarantor

The Guarantor is the one or more people who accept full responsibility for the work and/or the conduct of the study, had access to the data, and controlled the decision to publish. Tatsuya Fukami.

## Consent

We confirmed the consent.

## Provenance and peer review

Not commissioned, externally peer reviewed.

## Declaration of competing interest

The authors have nothing to disclose.
